# Cloning and Molecular Characterization of HSL and Its Expression Pattern in HPG Axis and Testis during Different Stages in Bactrian Camel

**DOI:** 10.3390/cimb44080259

**Published:** 2022-08-21

**Authors:** Jinghong Nan, Qi Wang, Qiu Yan, Jie Wang, Yong Zhang, Xingxu Zhao

**Affiliations:** 1 Gansu Key Laboratory of Animal Generational Physiology and Reproductive Regulation, Lanzhou 730070, China; 2 College of Veterinary Medicine, Gansu Agriculture University, Lanzhou 730070, China; 3 College of Life Science and Technology, Gansu Agriculture University, Lanzhou 730070, China

**Keywords:** Bactrian camels, HSL, cloning, testis, gonad axis

## Abstract

Hormone-sensitive lipase (HSL) is a key enzyme in animal fat metabolism and is involved in the rate-limiting step of catalyzing the decomposition of fat and cholesterol. It also plays an important regulatory role in maintaining seminiferous epithelial structure, androgen synthesis and primordial germ cell differentiation. We previously reported that HSL is involved the synthesis of steroids in Bactrian camels, although it is unclear what role it plays in testicular development. The present study was conducted to characterize the biological function and expression pattern of the HSL gene in the hypothalamic pituitary gonadal (HPG) axis and the development of testis in Bactrian camels. We analyzed cloning of the cDNA sequence of the HSL gene of Bactrian camels by RT-PCR, as well as the structural features of HSL proteins, using bioinformatics software, such as ProtParam, TMHMM, Signal P 4.1, SOPMA and MEGA 7.0. We used qRT-PCR, Western blotting and immunofluorescence staining to clarify the expression pattern of HSL in the HPG axis and testis of two-week-old (2W), two-year-old (2Y), four-year-old (4Y) and six-year-old (6Y) Bactrian camels. According to sequence analysis, the coding sequence (CDS) region of the HSL gene is 648 bp in length and encodes 204 amino acids. According to bioinformatics analysis, the nucleotide and amino acid sequence of Bactrian camel HSL are most similar to those of *Camelus pacos* and *Camelus* *dromedarius*, with the lowest sequence similarity with *Mus musculus*. In adult Bactrian camel HPG axis tissues, both HSL mRNA and protein expression were significantly higher in the testis than in other tissues (hypothalamus, pituitary and pineal tissues) (*p* < 0.05). The expression of mRNA in the testis increased with age and was the highest in six-year-old testis (*p* < 0.01). The protein expression levels of HSL in 2Y and 6Y testis were clearly higher than in 2W and 4Y testis tissues (*p* < 0.01). Immunofluorescence results indicate that the HSL protein was mainly localized in the germ cells, Sertoli cells and Leydig cells from Bactrian camel testis, and strong positive signals were detected in epididymal epithelial cells, basal cells, spermatocytes and smooth muscle cells, with partially expression in hypothalamic glial cells, pituitary suspensory cells and pineal cells. According to the results of gene ontology (GO) analysis enrichment, HSL indirectly regulates the anabolism of steroid hormones through interactions with various targets. Therefore, we conclude that the HSL gene may be associated with the development and reproduction of Bactrian camels in different stages of maturity, and these results will contribute to further understanding of the regulatory mechanisms of HSL in Bactrian camel reproduction.

## 1. Introduction

Animal breeding and animal husbandry are currently facing challenges in the context of global warming, with increasing land desertification and an increasing demand for sustainable meat and dairy. Whereas camelid species that can adapt to harsh climatic conditions could represent an appropriate response to this challenge, we selected the most abundant Bactrian camel in the country as the subject of our study. Bactrian camels are mainly found in central Asian countries, including China, Kazakhstan, Mongolia, Russia, northeastern Afghanistan, Uzbekistan and Crimea [1]. China has the largest population of Bactrian camels, mainly in Inner Mongolia, Xinjiang, Qinghai and Gansu. However, the number of Bactrian camel has decreased dramatically in recent years, mainly due to their long growth cycle and seasonal breeding, which is pronounced and strictly seasonal in male camels. Therefore, improving Bactrian camel productivity and breeding conservation is a pressing issue.

It is well-known that the hump of the Bactrian camel contains a large amount of adipose tissue [2], mainly comprising saturated and unsaturated fatty acids [3]. Hormone-sensitive lipase (HSL) is a rate-limiting enzyme of lipolysis that plays a decisive role in the synthesis and metabolism of fat. It hydrolyze triglycerides, diglycerol, monoglycerol and cholestenyl esters [4,5] is expressed not only in adipose tissue but also in several organs in mammals [6,7,8,9,10,11]. Studies have shown [12] that testicular cells are rich in unsaturated fatty acids, cholesterol and plasma bilayers, which are closely related to lipid metabolism. Some scholars have detected high expression of HSL in human spermatogenic tubules [13]. Casado et al. found that all HSL lipid substrates were strictly distributed in male germ cells [14]. In HSL knockout mice, dysfunctional lipid homeostasis in the testis leads to changes in plasma membrane lipid rafts [15], as well as interruption of spermatogenesis and sterility, despite normal steroid hormone levels and mating frequency [4,13,16,17]. Reports of HSL genes identified in previous studies concerning mammalian reproductive physiology have focused on humans [13] and mice [4,13,16,17], and to the best of our knowledge, few studies have been conducted on camelids, particularly Bactrian camels.

Therefore, in the present study, we used molecular biology to investigate the regulation of HSL in the reproductive physiology of Bactrian camels in order to understand the sequence characteristics of the Bactrian camel HSL gene and its role and expression pattern in the development of testis and the hypothalamic pituitary gonadal (HPG) axis. We first cloned the CDS coding sequence of the Bactrian camel HSL gene using PCR; then, we examined the expression profile and positive distribution of the HSL gene in various tissues of the Bactrian camel HPG axis and in testis at different stages of sexual maturation. These results will provide further insights into the regulatory mechanisms and biological roles of HSL in the reproductive physiology of the Bactrian camel.

## 2. Materials and Methods

### 2.1. Animals and Sample Collection

All samples were collected in strict accordance with the ethical guidelines approved by the Animal Care Commission of the College of Veterinary Medicine, Gansu Agriculture University under ethical code GSAU-Eth-LST-2021-003. Male camels at varying stages of development, namely two-week-old juvenile (2W, *n* = 3), two-year-old pre-sexual maturity (2Y, *n* = 3), four-year-old somatic maturity (4Y, *n* = 3) and six-year-old adulthood (6Y, *n* = 3), were included in the present study. Male camel samples were selected from Zhangye (Gansu province, China). Fresh HPG tissues (including hypothalamus, pituitary gland, pineal gland, testis and epididymis tissue) were collected from Bactrian camels at adult age (6Y). Tissue samples were washed with PBS to remove blood. A portion of the tissue was placed in liquid nitrogen for storage and later stored at −80 °C for RNA and protein extraction. The remaining tissue was fixed with 4% paraformaldehyde and used to prepare paraffin sections.

### 2.2. RNA Isolation and cDNA Synthesis

Total RNA was extracted from the HPG axis of Bactrian camels (hypothalamus, pituitary, pineal, testis and epididymis) and testicular tissues at different ages (2W, 2Y, 4Y and 6Y) with TRIzol reagent (TransGen Biotech, Beijing, China) according to the manufacturer’s instructions. Using a micronucleic acid assay, 1 μL of total RNA was taken from each sample to determine its concentration and mass. The RNA samples were then reverse-transcribed with a cDNA SYBR^®^ Green Premix Pro Taq HS qPCR kit (code AG1170; Accurate biology, Changsha, Hunan, China) according to the manufacturer’s instructions.

### 2.3. CDS Region Cloning of HSL Gene

PCR and qPCR primers were designed using Primer Premier 3.0 based on the predicted mRNA sequence of the HSL gene of the Bactrian camel from the National Center for Biotechnology Information (NCBI), and primers were synthesized by DynaScience (Xi’an, China) (Table 1). Bactrian camel fat cDNA was used as a template to amplify the CDS sequence of the HSL gene. Cloning was performed using a 50 µL PCR reaction system, which consisted of 25 µL of 2× Easy Taq PCR SuperMix (TransGen Biotech, Beijing, China), 1 µL of template, 1 µL of forward and reverse primers and 22 µL of ddH_2_O. The polymerase chain reaction was completed using under following cycling conditions: reaction at 95 °C for 3 min, followed by 40 cycles at 95 °C for 30 s, 55 °C for 30 s and 72 °C for 1 min. The PCR products were separated on 1% agarose gel, and the products were purified using a gel extraction kit and sequenced by DynaScience.

### 2.4. Bioinformatics Analysis

The BLAST algorithm (http://blast.ncbi.nlm.nih.gov/Blast.cgi accessed on 17 February 2022) was used to find homologous sequences, and TMHMM software (http://www.cbs.dtu.dk/services/TMHMM/ accessed on 31 March 2022) was used to predict the protein transmembrane region. The basic physical and chemical properties of HSL were analyzed with ProtParam software (http://web.expasy.org/protparam/ accessed on 2nd April 2022). The secondary structure of HSL protein was predicted by SOPMA software (https://npsa-prabi.ibcp.fr/cgi-bin/npsa_automat.pl?page=npsa_sopma.html accessed on 5 March 2022). Phyre2 software (http://www.sbg.bio.ic.ac.uk/phyre2/html/page.cgii?id=index accessed on 5 March 2022) was used to predict the tertiary structure. A phylogenetic tree was constructed using the neighbor-joining (NJ) method with MEGA 7.0 software (Institute for Genomics and Evolutionary Medicine, Temple University, Philadelphia, PA, USA). A distance method was applied to first construct a distance matrix; the elements in the matrix represent the distance between pairs of organisms. Different clustering methods were used to generate the phylogenetic tree, and the reliability of each branch was tested with the maximum-likelihood method (1000 bootstrap replications).

### 2.5. Quantitative Real-Time PCR (qPCR)

Relative expression levels of HSL in adult Bactrian camel HPG and testicular tissues at different ages were measured using qPCR. The qPCR primers were designed in Premier 3.0 and synthesized by Qinke Biotechnology Co (Xi’an, China). qPCR was performed with a LightCycler 96 real-time system (Roche, Basel, Switzerland), with aa 20 μL reaction volume: 1 μL cDNA template, SYBR premix Ex Taq™II. All reactions were run in triplicate. The 2^−∆∆Ct^ method was used to calculate the mRNA expression of HSL mRNA relative to GAPDH as a housekeeping gene [18].

### 2.6. Western Blot

The relative expression of HSL proteins in each tissue (hypothalamus, pituitary, pineal, testis and epididymis) were examined using Western blot. A radioimmunoprecipitation (RIPA) protein extraction kit (Solarbio, Beijing, China) and phenylmethanesulfonyl fluoride (PMSF) (Solarbio, Beijing, China) were used to homogenize and lyse the protein samples according to the manufacturer’s instruction. Protein concentration was determined using a BCA kit (Solarbio, Beijing, China). Protein samples were separated by 12% sodium dodecyl sulfate polyacrylamide gel electrophoresis (SDS–PAGE). The imprinted electrode was transferred to a PVDF membrane (Millipore CAT, Billerica, MA, USA), blocked in Tris-HCl buffer containing 2.5% (*w*/*v*) skim milk powder for 2 h and incubated overnight with mouse monoclonal antibody HSL (1:800, Abmart, Shanghai, China) at 4 °C. The membranes were washed with PBST and incubated with goat anti-mouse IgG/HRP antibody (1:5000, Beijing, China) (1:5000, Beijing, China) at 37 °C for 1.5–2 h. The membranes were washed with PBST; finally, the bands were scanned and exposed by Image-Pro Plus 6.0 (Media Cybernetics Co., Rockville, MD, USA). The experiment was conducted at least three times.

### 2.7. Histologic and Immunofluorescence Analysis

H&E staining was used to observe the morphology of HPG-axis tissues of adult Bactrian camel and testis tissues at different ages. The fixed tissue blocks were trimmed into a suitable size and washed with water for 24 h to washed away and fixed liquid in the tissue. The, moisture in the tissue block was removed by gradient alcohol, and samples were placed in xylene to make them transparent. The transparent tissue was placed in paraffin (Solarbio, Beijing, China) for embedding. The samples were cut into 5–8 μm thick slices with a slicing machine (Lecia, Weztlar, Germany). The slices were scalded in hot water and dried on a glass slide. The samples were then dewaxed with xylene, gradient-precipitated with ethanol and dyed with hematoxylin and eosin after rinsing with water [19]. For immunofluorescence detection, after routine dewaxing and hydration, the sections were heated with a microwave to repair the section antigens. Next, the slices were incubated with mouse anti–HSL antibody (1:120; abmart, Shanghai, China) overnight at 4^°^C, washed with PBS and incubated room temperature with FITC or ALEX-binding goat anti-mouse IgG (1:600; abcam, Cambridge, MA, USA). Next, slices were incubated with rabbit anti-AR antibody (1:110; abcam, Cambridge, MA, USA) overnight at 4 °C, washed with PBS and incubated with goat anti–rabbit IgG (1:600; Abcam, Cambridge, MA, USA). The nuclei were stained with 4,6-diamino-2-phenylindole (DAPI). Finally, digital images were captured using CaseViewer software (3DHISTECH, Budapest, Hungary).

### 2.8. Gene Ontology Analysis

Gene ontology analysis allows for annotation of gene products, including annotation of cell composition, biological processes and molecular functions. DAVID was used to enrich the GO function of the HSL gene. The number of differentially expressed genes contained in each item in GO was counted, and the enrichment score was calculated. The higher the enrichment score value, the more significant the degree of enrichment of the items in this GO. The protein–protein interaction (PPI) networks were constructed using the candidate proteins involved in sterol hormone biosynthesis with the STRING v 10.0 database (online, https://www.string-db.org/ accessed on 20 April 2022).

### 2.9. Statistical Analysis

The relative expression of HSL mRNA was calculated by the 2^−∆∆Ct^ method [18]. The band density value of the HSL protein was analyzed using one-way analysis of variance in SPSS 21.0 (SPSS Inc., Chicago, IL, USA). All data are shown in the form of bar charts as mean ± standard deviation (SD). *p* < 0.01 indicates that the difference was extremely significant, and *p* < 0.05 indicates that the difference was significant.

## 3. Results

### 3.1. Cloning and Sequence Analysis of Bactrian Camel HSL CDS

**Figure 1:**
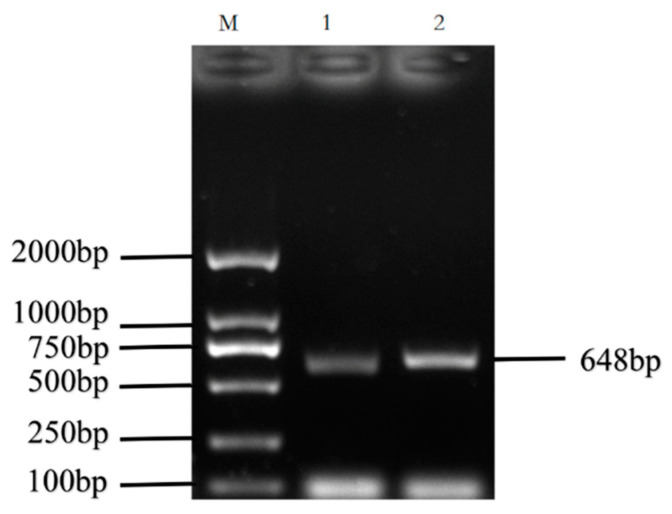
PCR amplification products of the Bactrian camel HSL coding sequence (CDS). M, DL2 000 marker; 1 and 2, PCR product.

**Figure 2:**
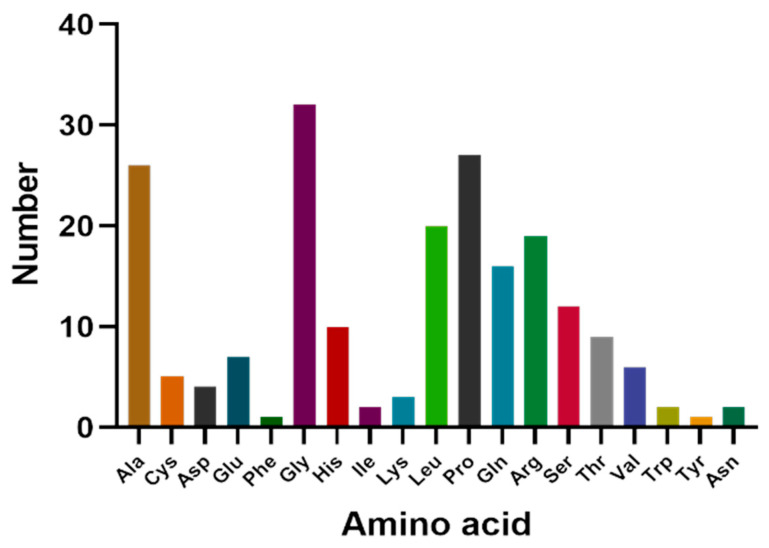
Amino acid composition of Bactrian camel HSL CDS region.

**Figure 3:**
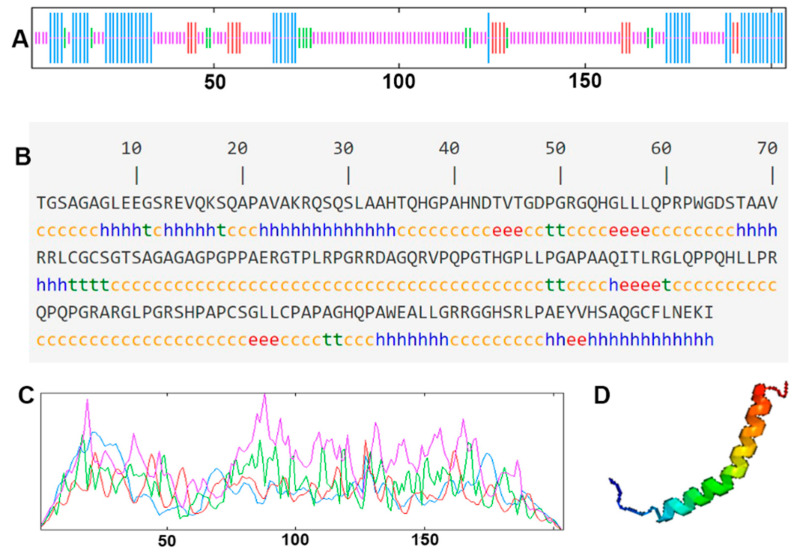
The secondary structure and tertiary structure of Bactrian camel HSL protein. (**A**,**C**) Secondary structure; different structures are represented by different color lines: blue, α helix; red, extended strand; green, β turn; purple, random coil. (**B**) Nucleotides and translated amino acid sequences of the CDS region of HSL. (**D**) Predictive tertiary molecular structure.

The cDNA of Bactrian camel adipose tissue was used as a template for PCR amplification, and a specific target fragment of 648 bp was obtained (Figure 1). Sequence analysis revealed an open reading frame (ORF) encoding a translation product of 204 amino acids. Because online software can be used to statistically analyze the secondary structure of proteins, no chemical analysis was carried out. The molecular formula of the protein encoded by the HSL gene was analyzed by ProtParam software and predicted to be C_897_H_1442_N_304_O_267_S_5_, with a relative molecular mass of 20.91749 kDa and a theoretical isoelectric point of 10.93, with 11 negatively charged amino acid residues (Asp + Glu) and 22 positively charged amino acid residues (Arg + Lys). The half-life of Bactrian camel HSL protein was 7.2 h, with a lipid factor of 59.31, an average hydrophilicity of −0.654, no transmembrane regions in the sequence and an instability index of 59.31. There were 34 potential phosphorylation sites, including 12 serine phosphorylation sites, 9 threonine phosphorylation sites and 1 tyrosine phosphorylation site. Therefore, we suggest that the HSL protein of Bactrian camels is a hydrophobically unstable, non-transmembrane protein. According to analysis of amino acid composition, Gly was the main amino acid (16.49%),followed by Pro (12.16%), Ala (9.46%), Leu (8.38%), Arg (7.57%), Gln (7.30%), Phe (0.49%) and Tyr (0.49%) (Figure 2). Secondary structure predictions revealed that the HSL protein consists of α-helices (51, 25%), β-turns (13, 6.37%), random coils (124, 60.78%) and extended chains (16, 7.8%) (Figure 3A–D). The tertiary structures of the HSL protein further spatially extended based on random coils, alpha helixes and beta turns (Figure 3B).

### 3.2. Homology Analysis and Evolutionary Relationships of the HSL Gene among Different Species

**Figure 4:**
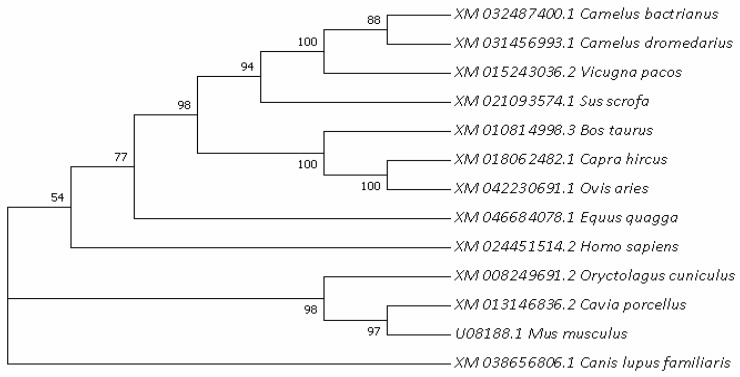
Evolutionary relationships analyzed based on nucleotide sequences of the HSL gene among different mammals.

In order to further understand the evolutionary relationships of the HSL gene, the phylogenetic trees of HSL in different species were constructed (Figure 4), with actrian camels clustered primarily with dromedaries and posteriorly with musculus. The results show that Bactrian camel HSL is most closely related to *Camelus* *dromedarius* and *Camelus pacos*, followed by *Sus scorfa* and cattle, showing the lowest similarity with musculus. The bootstrap value values of each branch shown Figure 4 were greater than 50%, and the vast majority were greater than 70%, indicating that the feasibility of each species branch was high.

### 3.3. Expression and Localization Analysis of HSL in Bactrian Camel HPG-Axis Tissues

**Figure 5:**
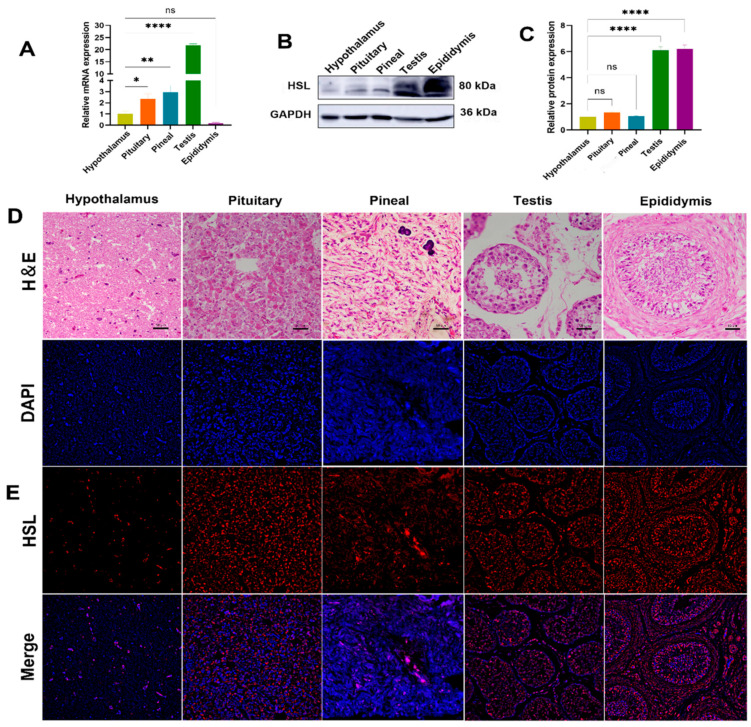
Expression and localization analysis of HSL in Bactrian camel HPG-axis tissues. (**A**) The expression level of HSL mRNA detected in Bactrian camel HPG-axis tissues by quantitative real-time PCR, with GAPDH as an internal control. Values represent mean ± SD, *n* = 3. * *p* < 0.05, ** *p* < 0.01, **** *p* < 0.001. (**B**) Western blot analysis of HSL protein and GAPDH for samples from HPG-axis tissues. (**C**) The expression density of detected HSL protein; data are expressed as the mean ± SD, **** *p* < 0.001. (**D**) Morphology was identified by H&E staining. (**E**) Analysis of intracellular HSL protein localization in Bactrian camel HPG-axis tissues by immunofluorescence.

The expression levels of HSL mRNA and protein in HPG-axis tissues (hypothalamus, pituitary, pineal, testis and epididymis) of Bactrian camels were determined by qPCR and Western blot. qPCR results show that the expression of HSL mRNA differ significantly among tissues of the HPG axis, with the highest expression levels of HSL mRNA in testis tissues, followed by pineal tissues, with significantly lower expression in pituitary, hypothalamus and epididymis tissues and the lowest level of expression in epididymis tissues (Figure 5A). Western blotting results show that expression differs between tissues, with low levels of expression detected in hypothalamus, pituitary and pineal tissues and high levels of expression detected in the testis and epididymis (Figure 5B,C). Immunofluorescence results show that HSL is distributed on various tissues of the HPG axis. The hypothalamus was found to contain various forms of glial cells, and HSL was expressed on glial cells. The pituitary gland was found to be adenopituitary and consisted of eosinophils, basophils and suspensory cells, with HSL distributed in suspensory cells. In pineal tissues, HSL was mainly expressed on pineal cells and glial cells. In testicular tissues, HSL was expressed on spermatogonia, Leydig cells and Sertoli cells. In epididymal tissues, HSL was expressed on epithelial cells, basal cells, spermatocytes and smooth muscle cells (Figure 5D,E).

### 3.4. Expression and Localization of HSL in the Testis of Bactrian Camels at Different Ages

**Figure 6:**
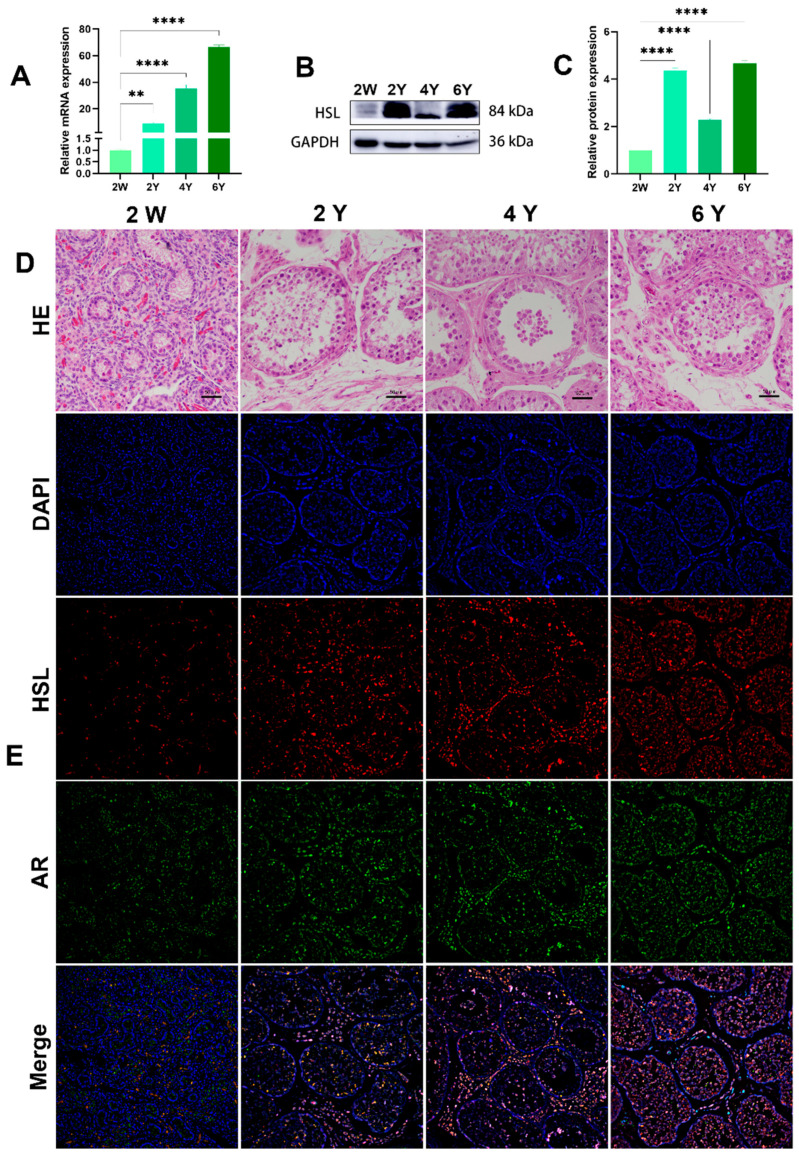
Expression and localization of HSL in Bactrian camel testicular tissues at different ages. (**A**) Expression levels of HSL mRNA in testis at different ages visualized by qRT-PCR. Values represent mean ± SD. ** *p* < 0.01, **** *p* < 0.001. (**B**) Western blot and (**C**) expression density analysis of HSL and GAPDH protein expression levels in samples at different ages. Values represent mean ± SD. **** *p* < 0.001. (**D**) Morphology was identified by H&E staining. (**E**) Analysis of intracellular HSL protein localization in testis by immunofluorescence.

The *HSL* mRNA level was detected by quantitative real-time PCR (qRT-PCR) in Bactrian camel testicular tissues at different ages. Protein levels in the testis at different ages were analyzed by Western blotting. GAPDH, as a housekeeping gene control, showed clear mRNA-positive signals in all samples. The qRT-PCR results show that HSL mRNA was most highly expressed in 6Y testis, followed by 4Y and 2Y, with the lowest level of mRNA expression level in 2W testis (Figure 6A). Western blot results suggest that HSL protein expression was abundant in testis tissues at different ages, with the lowest level of HSL protein expression in the 2W and significantly increased expression in the 2Y group, 4Y group and 6Y group (Figure 6B,C). Immunofluorescence staining showed that 2W testis had undifferentiated seminiferous tubules with an indistinct shape, and the basement membrane of the varicose ducts was initially formed with a few ovoid Sertoli cells mixed with germ cells. In the 2Y, 4Y and 6Y testis, the testicular seminiferous tubules were further developed, and the germ cells were stratified, including spermatogonia, primary spermatocytes, secondary spermatocytes and spermatozoa in increased numbers were. The positive signals of androgen receptor AR (Sertoli cell and Leydig cell marker protein) and HSL protein at were detected at different developmental stages (Figure 6D,E). A small amount of HSL protein expression was detected in the germinal tubules and mesenchyme of the 2W testis, and the signals of HSL on germ cells, Sertoli cells and Leydig cells were enhanced with increased maturity.

### 3.5. Functional Analyses of Bactrian Camel HSL in Steroid Hormone Synthesis and Metabolism

**Figure 7:**
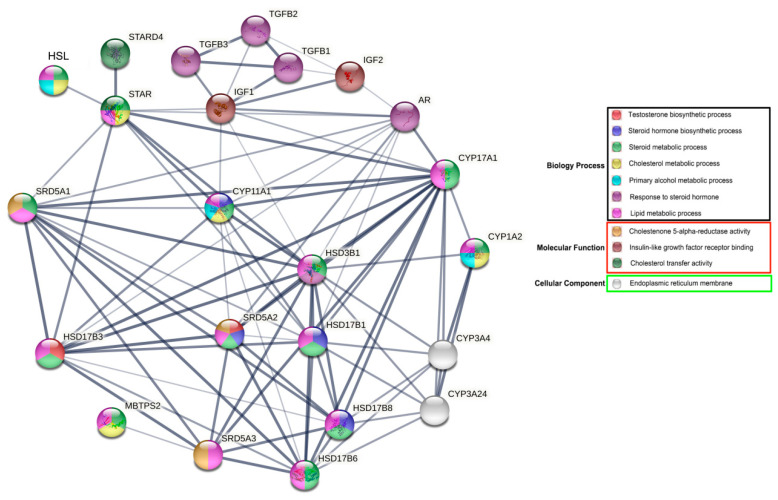
Network of GO terms and associated proteins of Bactrian camel HSL in steroid hormone synthesis and metabolism.

The results of gene ontology (GO) functional enrichment analyses show that HSL is directly or indirectly involved in various biological processes associated with reproductive hormone and lipid metabolism, particularly in steroid hormone metabolism. HSL may directly regulate steroid hormone synthesis acute regulator protein (STAR). In addition, HSL indirectly regulates the androgen receptor (AR) via important rate-limiting enzymes or cytokines that regulate synthesis and metabolism, including steroid 5 alpha–reductase 1 (SRD5A1); cytochrome P450, family 11, subfamily A and polypeptide 1 (CYP11A1); 17-β hydroxysteroid dehydrogenase 3 (HSD17β3); steroidogenic acute regulatory protein-related lipid transfers 4 (STARD4); steroid 5 alpha-reductase 2 (SRD5A2); and hydroxy-delta-5-steroid dehydrogenase–3 beta (HSD3B1). Additionally, HSL may participate indirectly in insulin–like growth factor–1/2 (IGF–1/2) and β-transforming growth factors (TGFBs) (Figure 7). Therefore, we hypothesized that HSL may directly or indirectly regulate steroid hormone synthesis and metabolism.

## 4. Discussion

In this study, the CDS sequence of Bactrian camel HSL was cloned via PCR, and physicochemical properties of HSL were analyzed. The results suggest that HSL protein has 12 potential serine (Ser) kinase phosphorylation sites, 9 potential threonine (Thr) kinase phosphorylation sites and 1 tyrosine (Tyr) kinase phosphorylation sites and that phosphorylation of the protein is closely related to HSL activation [20]. The catalytic region and lipid-binding region of HSL are known to have hydrophobic properties, which is consistent with the prediction results obtained in this study. In addition, the HSL CDS sequence of Bactrian camel has a high degree of homology (no less than 90%) with other published mammalian HSL sequences from the NCBI. Previous studies have suggested that HSL may be involved in spermatogenesis and androgen synthesis in the testis [13,16,17,21,22]. However, little has been reported about the HSL function in Bactrian camels, and the mechanism of regulation is unknown. Androgens are primarily regulated by the reproductive axis (hypothalamus–pituitary–testis axis) and are responsible for hormonal regulation and modulation of reproductive function and behavior in males. Therefore, we explored the expression pattern of HSL in the reproductive axis. The results suggest that HSL is highly expressed in testis. To further investigate the regulation function in the reproductive system of Bactrian camels, we detected the expression level of HSL at different stages the testis. These results suggest that the expression of HSL is upregulated with age during the development of Bactrian camel testis, which is consistent with the findings reported by Holst et al. [23].

It was previously reported that inactivation of HSL does not lead to severe lipid metabolism disorders and that there is another lipase in fat with similar catalytic activity to that of HSL: adipose triglyceride lipase (ATGL) [24], which regulates fatty acids in vivo, and is a triglyceride hydrolase. However, HSL mainly hydrolyzes diglycerides and supports specific biological functions, such as spermatogenesis [17]. Spermatogenesis is a complex process controlled by the interaction of multiple cells in the testis, including macrophages, peritubular cells, Leydig cells (LCs), Sertoli cells (SCs) and germ cells [25]. Mediated by hormones such as testosterone, the germinal epithelium matures to produce spermatozoa, which later transit through the head, body and tail of the epididymis to gain the ability to fertilize. During this process, there is a close association between neutral fatty acids, adenylate cyclase within the epididymis and phospholipid fatty acid content. It has been suggested that HSL is not present in Leydig cells [26]. However, it has also been found that the HSL gene can be detected in guinea pig [27] and human [13] mesenchymal cells and sperm and is positively correlated with testosterone levels, suggesting that androgen production may be involved, which is consistent with the results of our study. Hermo et al. [28] found that HSL is the only esterase that can break down cholesterol esters in the testis, and tests can demonstrate that HSL knockdown results in the accumulation of cholesterol lipids in the testis. Therefore, the substrate for HSL action is lipid droplets, and the presence of lipid droplets in both Sertoli cells and testicular Leydig cells suggest that HSL may be involved in male Bactrian camel Sertoli cells and testicular Leydig cells during spermatogenesis and androgen synthesis.

GO enrichment analysis showed that HSL directly or indirectly interacts with proteins related to steroid hormone biosynthesis, especially STAR, HSD3B, SRD5A1 and IGF1, the rate-limiting enzymes and regulatory factors of steroidogenesis. For example, under the action of STAR protein, cholesterol is transferred from mitochondria to the endometrium, further acting on the biosynthesis of steroid hormones in steroidogenic cells [29]. Testosterone is converted into 5–dihydrotestosterone by SRD5A in steroidogenic tissues and peripheral tissues [30], indicating that HSL participates in the regulation of steroid hormone synthesis and secretion by regulating the expression levels of STAR, HSD3B, IGF1and SRD5A1. HSL is also expressed in the adrenal gland, which is mainly involved in the regulation of cholesterol kinase activity. For example, the activity of cholesterol kinase in the adrenal gland of mice with HSL gene deletion is less than that of normal mice, showing obvious lipid accumulation, resulting in a decrease in the production of adrenocorticotropic hormones [31], suggesting that HSL provides cholesterol and other precursors for the synthesis of steroid hormones.

In this study, the expression pattern, localization, potential function and regulatory network of HSL were described, although the specific regulatory role of HSL in hormone metabolism requires further study. The above results will contribute to improved understanding the reproductive physiology of male Bactrian camels and provide a theoretical basis for future studies on the reproductive performance of Bactrian camels.

## 5. Conclusions

In summary, this is the first study to characterize the CDS sequence of the HSL gene in Bactrian camels and to explore its expression pattern and reproduction-related function. The prediction-length CDS of the Bactrian camel *HSL* gene is 648 bases and encodes 204 amino acids. It is highly homologous to the nucleotide sequences of other Camelus species. HSL mRNA and protein are mainly expressed in Bactrian camel testis and epididymis, with an age-dependent increase in expression pattern in testis and low levels of expression in hypothalamus, pituitary and pineal tissues. HSL may interact directly or indirectly with steroid hormone biosynthesis-related proteins. Based on these findings, we suggested that HSL may be involved in the regulatory mechanism of steroid hormones and androgen synthesis in Bactrian camels through the steroid synthesis pathway.

## Figures and Tables

**Table 1:** Primers sequence information of mouse genes for PCR.

Gene	Sequence (5′-3′)	Tm (°C)	Length/bp	GenBank No.
*HSL*	ATGGAATCGGCCAGAGAAACG CTAGTTACTAGTTACGTAGAAACAGCCCT	60	648	XM_032487400.1
*HSL*	AACCGCCGCAGCATCTT CCCTCGTCGCCCTCAAA	58	155	XM_045510514.1
*GAPDH*	AACATCATCCCTGCTTCTACC ATGCCTGCTTCACTACCTTCT	56	184	NM_001357943.2

## Data Availability

Not applicable.

## References

[1] Mirzaei F. (2012). Production and trade of camel products in some Middle East countries. J. Agric. Econ. Dev..

[2] Sbihi H.M., Nehdi I.A., Al-Resayes S.I. (2013). Characterization of Hachi (*Camelus dromedarius*) fat extracted from the hump. Food Chem..

[3] El-Anany A.M., Ali R.F.M. (2018). Physicochemical characteristics of binary mixtures of camel hump fat and citrus seed oil. Riv. Ital. Sostanze Grasse.

[4] Holm C., Kirchgessner T.G., Svenson K.L., Fredrikson G., Nilsson S., Miller C.G., Shively J.E., Heinzmann C., Sparkes R.S., Mohandas T. (1988). Hormone-sensitive lipase: Sequence, expression, and chromosomal localization to 19 cent-q13.3. Science.

[5] Holm C. (2003). Molecular mechanisms regulating hormone-sensitive lipase and lipolysis. Biochem. Soc. Trans..

[6] Varlamov O., Chu M.P., McGee W.K., Cameron J.L., O’Rourke R.W., Meyer K.A., Bishop C.V., Stouffer R.L., Roberts C.T. (2013). Ovarian cycle-specific regulation of adipose tissue lipid storage by testosterone in female nonhuman primates. Endocrinology.

[7] Jaffer I., Riederer M., Shah P., Peters P., Quehenberger F., Wood A., Scharnagl H., März W., Kostner K.M., Kostner G.M. (2012). Expression of fat mobilizing genes in human epicardial adipose tissue. Atherosclerosis.

[8] Jocken J.W., Goossens G.H., Boon H., Mason R.R., Essers Y., Havekes B., Watt M.J., van Loon L.J., Blaak E.E. (2013). Insulin-mediated suppression of lipolysis in adipose tissue and skeletal muscle of obese type 2 diabetic men and men with normal glucose tolerance. Diabetologia.

[9] Hołysz M., Trzeciak W.H. (2015). Hormone-sensitive lipase/cholesteryl esterase from the adrenal cortex-structure, regulation and role in steroid hormone synthesis. Postepy Biochem..

[10] Roduit R., Masiello P., Wang S.P., Li H., Mitchell G.A., Prentki M. (2001). A role for hormone-sensitive lipase in glucose-stimulated insulin secretion: A study in hormone-sensitive lipase-deficient mice. Diabetes.

[11] Wang F., Chen Z., Ren X., Tian Y., Wang F., Liu C., Jin P., Li Z., Zhang F., Zhu B. (2017). Hormone-sensitive lipase deficiency alters gene expression and cholesterol content of mouse testis. Reproduction.

[12] Chap H. (2016). Forty five years with membrane phospholipids, phospholipases and lipid mediators: A historical perspective. Biochimie.

[13] Blaise R., Guillaudeux T., Tavernier G., Daegelen D., Evrard B., Mairal A., Holm C., Jégou B., Langin D. (2001). Testis hormone-sensitive lipase expression in spermatids is governed by a short promoter in transgenic mice. J. Biol. Chem..

[14] Casado M.E., Pastor O., Mariscal P., Canfrán-Duque A., Martínez-Botas J., Kraemer F.B., Lasunción M.A., Martín-Hidalgo A., Busto R. (2013). Hormone-sensitive lipase deficiency disturbs the fatty acid composition of mouse testis. Prostaglandins Leukot. Essent. Fat. Acids.

[15] Casado M.E., Huerta L., Ortiz A.I., Pérez-Crespo M., Gutiérrez-Adán A., Kraemer F.B., Lasunción M., Busto R., Martín-Hidalgo A. (2012). HSL-knockout mouse testis exhibits class B scavenger receptor upregulation and disrupted lipid raft microdomains. J. Lipid Res..

[16] Haemmerle G., Zimmermann R., Hayn M., Theussl C., Waeg G., Wagner E., Sattler W., Magin T.M., Wagner E.F., Zechner R. (2002). Hormone-sensitive lipase deficiency in mice causes diglyceride accumulation in adipose tissue, muscle, and testis. J. Biol. Chem..

[17] Osuga J., Ishibashi S., Oka T., Yagyu H., Tozawa R., Fujimoto A., Shionoiri F., Yahagi N., Kraemer F.B., Tsutsumi O. (2000). Targeted disruption of hormone-sensitive lipase results in male sterility and adipocyte hypertrophy, but not in obesity. Proc. Natl. Acad. Sci. USA.

[18] Livak K.J., Schmittgen T.D. (2001). Analysis of relative gene expression data using real-time quantitative PCR and the 2(-Delta Delta C(T)) Method. Methods.

[19] Otali D., Fredenburgh J., Oelschlager D.K., Grizzle W.E. (2016). A standard tissue as a control for histochemical and immunohistochemical staining. Biotech. Histochem. Off. Publ. Biol. Stain. Comm..

[20] Olsson H., Strålfors P., Belfrage P. (1986). Phosphorylation of the basal site of hormone-sensitive lipase by glycogen synthase kinase-4. FEBS Lett..

[21] Wang F., Ren X.F., Chen Z., Li X.L., Zhu H.J., Li S., Ou X.H., Zhang C., Zhang F.X., Zhu B.C. (2019). The N-terminal His-tag affects the triglyceride lipase activity of hormone-sensitive lipase in testis. J. Cell. Biochem..

[22] Chung S., Wang S.P., Pan L., Mitchell G., Trasler J., Hermo L. (2001). Infertility and testicular defects in hormone-sensitive lipase-deficient mice. Endocrinology.

[23] Holst L.S., Langin D., Mulder H., Laurell H., Grober J., Bergh A., Mohrenweiser H.W., Edgren G., Holm C. (1996). Molecular cloning, genomic organization, and expression of a testicular isoform of hormone-sensitive lipase. Genomics.

[24] Zimmermann R., Strauss J.G., Haemmerle G., Schoiswohl G., Birner-Gruenberger R., Riederer M., Lass A., Neuberger G., Eisenhaber F., Hermetter A. (2004). Fat mobilization in adipose tissue is promoted by adipose triglyceride lipase. Science.

[25] Majumdar S.S., Bhattacharya I. (2013). Genomic and post-genomic leads toward regulation of spermatogenesis. Prog. Biophys. Mol. Biol..

[26] Holst L.S., Hoffmann A.M., Mulder H., Sundler F., Holm C., Bergh A., Fredrikson G. (1994). Localization of hormone-sensitive lipase to rat Sertoli cells and its expression in developing and degenerating testes. FEBS Lett..

[27] Mairal A., Melaine N., Laurell H., Grober J., Holst L.S., Guillaudeux T., Holm C., Jégou B., Langin D. (2002). Characterization of a novel testicular form of human hormone-sensitive lipase. Biochem. Biophys. Res. Commun..

[28] Hermo L., Chung S., Gregory M., Smith C.E., Wang S.P., El-Alfy M., Cyr D.G., Mitchell G.A., Trasler J. (2008). Alterations in the testis of hormone sensitive lipase-deficient mice is associated with decreased sperm counts, sperm motility, and fertility. Mol. Reprod. Dev..

[29] Stocco D.M. (2001). StAR protein and the regulation of steroid hormone biosynthesis. Annu. Rev. Physiol..

[30] Yazawa T., Inaba H., Imamichi Y., Sekiguchi T., Uwada J., Islam M.S., Orisaka M., Mikami D., Ida T., Sato T. (2021). Profiles of 5α-Reduced Androgens in Humans and Eels: 5α-Dihydrotestosterone and 11-Ketodihydrotestosterone Are Active Androgens Produced in Eel Gonads. Front. Endocrinol..

[31] Li H., Brochu M., Wang S., Rochdi L., Côté M., Mitchell G., Gallo-Payet N.J.E. (2002). Hormone-sensitive lipase deficiency in mice causes lipid storage in the adrenal cortex and impaired corticosterone response to corticotropin stimulation. Endocrinology.

